# The Relation of the Veterinarian to His Profession and the Public1Presented at the A. V. M. A. Meeting, Atlantic City, September, 1901.

**Published:** 1901-12

**Authors:** Walter Shaw

**Affiliations:** Dayton, Ohio


### THE RELATION OF THE VETERINARIAN TO HIS PROFESSION AND THE PUBLIC.[1]

By Walter Shaw, V.S.,
DAYTON, OHIO.

Because of our anxiety to solve the numberless questions which press for solution, and in our eagerness to establish plausible theories, we lose sight of the things which are of the most cardinal and practical importance to our profession.

The time is at hand when veterinary science is recognized as being a chief factor in the development of our civilization. The public more and more regards our profession as a necessity, and the demand for the service of good veterinarians is correspondingly increasing.

This organization has quite a large constituency, and it behooves

[1] Presented at the A. V. M. A. Meeting, Atlantic City, September, 1901.

us as individuals to improve the conditions on which the best development of veterinary science depends. In many ways we all stand obligated both to our profession and to the public, but these obligations are not infrequently overlooked. The ethical principle of unselfishness must be recognized. Knowledge, courtesy, agreement, co-operation—all are pertinent to the subject, and essential to the highest interests of this honorable calling.

With your permission I shall point out some wrongs to be righted and certain conditions to be ameliorated. The first is the disagreement of veterinarians in their diagnosis in certain cases. It is not extraordinary for their verdicts to conflict in the courts. For instance, this case: A horse has been sold under a warranty; in a few days the animal becomes lame, and the purchaser brings suit for damages. The veterinarian employed by the warrantee testifies in court that he has carefully examined the horse and discovered that the animal was suffering from chronic navicular disease; that it has been latent in the foot for many months, and now for the first time gives visible evidence of its presence, thus proving the unsoundness of the animal on the day of sale when the warranty was issued.

Contrariwise, the veterinarian in behalf of the warrantor, takes oath that he has made a deliberate examination, and decides that the horse is disabled in the shoulder. This veterinarian ascribes the lameness to many possible causes—slipping, stepping on a rolling stone, etc.; but he insists that the horse was sound when sold. Such instances are deplorable, and work mightily against the interests of the veterinary profession.

In London there is a parallel case on record in authentic annals in which two veterinarians pronounced a horse, recently purchased, to be a roarer. The veterinarian under the employ of the seller officially testified in the court that the horse was entirely sound, basing such a verdict on an examination of the throat by external manipulation only. As a consequence, the judge instructed the jury not to take into consideration the testimony of the latter veterinarian, because it was not pertinent to the case at hand. The degree of distrust created in the minds of the judge, jury, and people is incalculable. When members of a scientific profession so widely disagree as in the cases just cited, we must not be surprised when some decry our profession and proscribe veterinarians.

It frequently occurs that when a veterinarian locates in some city the proprietor of a boarding-stable who owns a few horses approaches him and offers him all the patronage he commands, on the condition that the veterinarian's service for his own horses be rendered gratis. Any wise and resolute doctor will immediately give such a man to understand that he is not buying practice, but that he expects to merit it, and the result of such a method will be an enlarged patronage, duly compensated. Any other method is disappointing, and must terminate in humiliation, loss of confidence, and financial collapse.

Again, a smooth-tongued horse-dealer praises the ability of the veterinarian; he asks him to recommend a certain horse in order that a sale may be effected, and then thoughtlessly accepts a proffered fee. In the course of a few weeks another horse with a blemish is brought, and the same favor is asked. After an examination the veterinarian refuses to pronounce him sound on account of a spavin, defective vision, or some ailment which interferes with the animal's usefulness. When he accepted the fee from the dealer his motives may have been unquestioned; but he is nevertheless defenceless because he has thoughtlessly placed himself under obligations to the dealer. The public would be justified in thinking that the doctor was in the "market," and that his decision relative to the soundness of an animal depended on the price offered.

A number of facts which come within the range of my personal knowledge might be cited, but the number instanced will suffice.

Another thing suggests itself here, and it is a condition which demands recognition, viz., the opportunities, demands, and sphere of usefulness for veterinarians are growing greater each successive year.

In the march of progress we must not linger in the rear, but be well versed in the sciences, skilled in surgery, and practical in methods. Serious responsibility rests upon us to-day as never before. Veterinarians who are employed by the State or local boards of health should render no definite verdict until they are assured that the animal, carcass, or milk is fit for human food. The public is necessarily dependent upon our profession for knowledge as to the exact condition of the meat-supply and milk-supply; consequently, they should conduct their examinations in such a manner as to increase the confidence of consumers in the profession. Especially should veterinarians who are employed by the State be scientific, professional, skilful, and accurate in their diagnosis of those diseases which affect both man and beast. The degree of responsibility placed upon inspectors and the profession, collectively considered, can only be realized when we remember that public health and the large live-stock interests of this country look to us for protection. The foreign demand for our live-stock, meat, and dairy products is regulated largely by the ability and accuracy of veterinarians. Here, again, the most glaring and humiliating disagreement is often to be observed.

It is not unusual to see on record an instance where the board of health prosecutes a man or firm for selling diseased meat or milk from diseased cows. When the case appears for trial in court the veterinarians under the employ of the board and defendant testify, under oath, that they made a careful examination of the products, and their conclusions directly conflict. It occurs to me that not infrequently the veterinarian in such cases is actuated by the same purpose as the attorney, viz., to acquit the client.

For emphasis, I repeat: The producer and consumer are dependent for protection upon our profession, and they demand (and have the right to demand) that veterinarians be accurate in their diagnosis, correct in their methods, just in their decisions, and conscientious in everything. So long as veterinarians who claim to be scientific give conflicting evidence and testimony from the witness-stand in our courts of justice and law, so long will our profession be held in low repute in some quarters. Surely such men have not a just and proper conception of what they owe to the public; neither do they realize the relations which they sustain to veterinary science.

No matter by whom subpœnaed, inspectors or veterinarians should render only such testimony as is tenable. If, after consultation and examination, they widely differ in their opinions, then no definite decision should be made, as it would but militate against the profession.

This point should be accentuated until its gravity is duly appreciated. In this day of unparalleled intellectual activity we cannot afford to be careless or uncertain in any particular. Too much is involved to sacrifice the truth for favor from any quarter. The exorbitant prices paid for our best horses, cattle, sheep, and hogs by foreign buyers must be attributed to veterinarians, who have shown their ability both to inspect meats and control contagious diseases at abattoirs, quarantines, and shipping-points. It is conceded on every hand by owners that we save them millions of dollars annually by exterminating such diseases from their herds and flocks.

There are various other facts which have a tendency to lower the standard of our profession against which public and professional sentiment should be aroused. There are two States which have granted charters for the establishment of veterinary colleges to men who are simply quacks—utterly incapable. We also have a college which granted diplomas to students who were in attendance but one summer. Another circulated letters and distributed cards soliciting students, on which the following humiliating statement was printed: "The lowest fee and shortest term." Such colleges fifty years ago would have been a reproach to the profession. It is as surprising as it is alarming to veterinary science that those in authority in those States should be so unmindful of the rights of the people as to grant charters to such men for the establishment of colleges in which young men are to be scientifically trained for such an important profession.

In saying this we are not unmindful of the fact that it is an honor to graduate from the majority of veterinary colleges, but we are now presenting some of the facts which have a tendency to lower rather than to raise the standard of our profession.

I want to cite to you another matter which has a baleful bearing on our vocation. There are some veterinarians who associate with and recommend travelling empirics who claim to have wide experience in surgical and dental work, but in reality they are inferior and very incompetent men.

Others mislead and deceive the public by indorsing certain chemical mixtures of secret formulas. Every possible influence should be brought to bear upon such veterinarians, that they may be dissuaded from recommending any medical mixture (whose ingredients are unknown) as if it were some panacea for all the ills to which equine flesh is susceptible.

It does not come within the limitations of my subject to suggest the remedies for these evils, but we suggest that serious effort be made to solve them.

Of this we are assured: that we must raise the standard for matriculation and have the most thorough training in our colleges, strictly business methods, and conscientious veterinarians before our profession will receive the public confidence and social recognition to which it is justly entitled.

---

Dr. E. K. Stretch, of Hoboken, N. J., is largely interested in the Metropolitan Dairy Company, and looks after the sanitary surroundings, etc., of the dairies whose products they receive.

